# The Transcriptional Programme of Human Heart Valves Reveals the Natural History of Infective Endocarditis

**DOI:** 10.1371/journal.pone.0008939

**Published:** 2010-01-28

**Authors:** Marie Benoit, Franck Thuny, Yannick Le Priol, Hubert Lepidi, Sonia Bastonero, Jean-Paul Casalta, Frédéric Collart, Christian Capo, Didier Raoult, Jean-Louis Mege

**Affiliations:** 1 Unité de Recherche sur les Maladies Infectieuses Transmissibles et Emergentes, Centre National de la Recherche Scientifique Unité Mixte de Recherche 6236, Université de la Méditerranée, Faculté de Médecine, Marseille, France; 2 Service de Cardiologie, Hôpital de la Timone, Marseille, France; 3 Relation Hôte-Parasites, Pharmacologie et Thérapeutique, Institut de Médecine Tropicale du Service de Santé des Armées, Marseille, France; 4 Service de Chirurgie Cardiaque, Hôpital de la Timone, Marseille, France; Leiden University Medical Center, Netherlands

## Abstract

Infective endocarditis (IE) is an infectious disease that is mainly caused by *Staphylococcus aureus* and *Streptococcus* sp. It usually leads to valvular destruction and vegetation formation. Its pathophysiology is badly understood and likely involves immune and coagulation systems with close interactions with the microorganism. Our objective was to evaluate host response by comparing transcriptional profiles of cardiac valves from IE patients with controls. Hierarchical clustering revealed a signature of IE consisting of 146 genes. Among the 89 up-regulated genes, we identified two genes strongly associated with IE: metalloproteinase 12 (MMP-12) and aquaporin-9, a member of the aquaglyceroporin membrane channel family. The up-regulation of MMP-12 gene is strengthened by the down-modulation of the gene encoding its inhibitor TIMP3. In addition, MMP-12 was expressed in macrophages infiltrating EI valves. We also found that aquaporin-9 was expressed in endothelial cells lining neo-vessel lumen, suggesting that aquaporin-9 might be associated with neovascularization of infected valves leading to tissue oedema secondary to the inflammatory process. The Gene Ontology annotation and the resulting functional classification showed that most up-regulated genes account for recruitment of inflammatory cells in vegetations, angiogenesis and remodelling of endocardium tissue. A network analysis confirmed the involvement of molecules related to the remodelling of endocardium tissue and angiogenesis in IE. It also evidenced the role of caspases, especially that of caspase-9 and intrinsic apoptotic pathway in IE. Based on this study we propose a scenario for the natural history of IE in humans. Some parameters identified in this work could become tools for measuring the disease activity and should be tested as biomarkers for diagnosis or prognosis assessment in future studies.

## Introduction

Infective endocarditis (IE) is a rather common disease causing high morbidity and mortality despite the availability of antimicrobial agents and cardiac surgery. Usually, IE is diagnosed by the culture of microorganisms (mainly *Staphylococcus aureus* and *Streptococci* sp.) from blood and echocardiographical or histological detection of vegetations. This lesion results from the formation of a coagulum consisting of plasma and platelet proteins [1]. Pathogens associated with IE bind the coagulum and colonize the lesion [1]–[3]. Most of the studies on IE pathophysiology have been obtained in experimental animals and show that the recruitment of circulating cells including neutrophils and monocytes is secondary to tissue reorganization and bacterial colonization [4], [5]. Cell recruitment leads to the release of cytokines and procoagulant factors and, consequently, to the enlargement of the vegetation. In addition, whereas normal heart valves are not vascularized, because of the expression of the anti-angiogenic factor chondromodulin-1, IE is associated with neo-vascularization [6]. Infecting pathogens survive inside vegetations by avoiding host defences, and the final step of IE consists of the dissemination of septic embols to distant organs and the destruction of valve tissues [7]. To investigate this complex host response, we studied the whole transcriptional activity of patient valves in the attempt to identify the major molecular pathways involved in IE pathophysiology. We consequently propose a scenario for the natural history of IE in humans and also suggest that some parameters identified in this work could become tools for measuring the disease activity and should be tested as biomarkers for diagnosis or prognosis assessment in future studies.

## Results and Discussion

**Figure 1:**
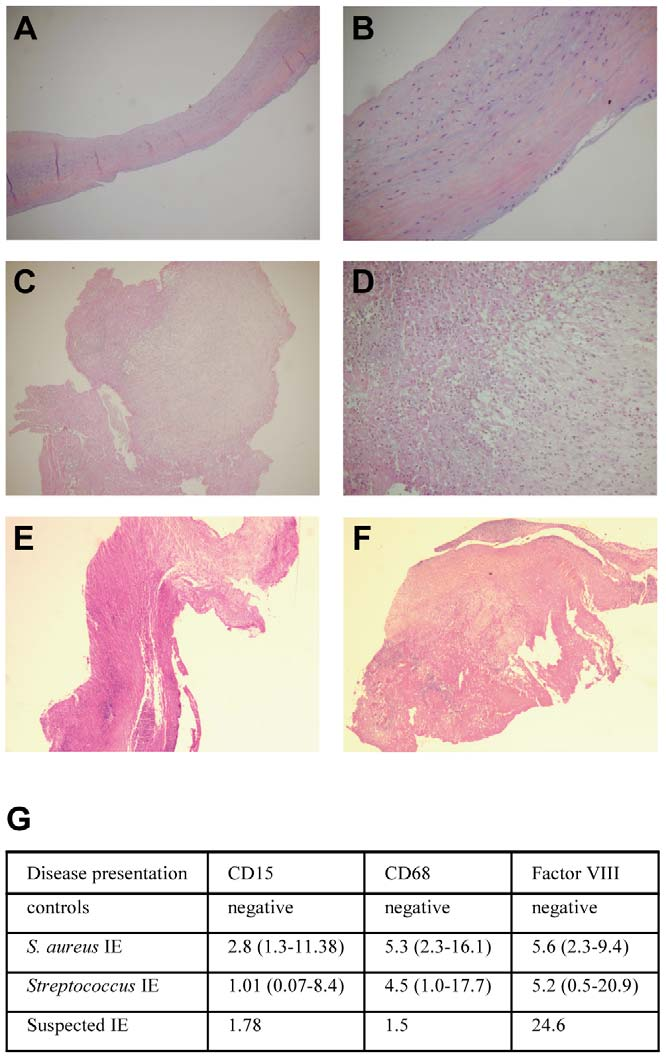
Histological analysis of cardiac valves. (A–F) Samples of cardiac valves from controls (A, B) and patients with IE due to *Streptococcus* (C, D) or *S. aureus* (E, F) were stained with haematoxylin-eosin-saffron. Representative micrographs are shown. Note the absence of vegetations and inflammatory infiltrates in controls. In IE patients, inflammatory infiltrates are mainly developed on the surface of the cusp of valves, within the vegetations. Left and right, ×25 and ×100 magnifications, respectively. (G) Valve sections were analyzed by immunohistochemistry and quantitative image analysis to determine the expression of CD15, CD68 and Factor VIII. The normalized results are expressed as the percentage of valve sample area covered by neutrophils, macrophages and endothelial cells, respectively. They represent the mean values, and the minimum and the maximum are shown in parentheses.

As IE is histologically characterized by infiltrates of inflammatory cells and neo-angiogenesis, we studied the expression of CD15 and CD68 as markers of neutrophils and macrophages, respectively, and Factor VIII as an angiogenesis marker. In valves from controls, no neutrophil, macrophage and factor VIII-expressing cells were detected (Fig. 1A–B). In contrast, the valves from patients with IE due to *Streptococcus* (Fig. 1C–D) or *S. aureus* (Fig. 1E–F) expressed CD15, CD68 and Factor VIII. CD15 represented 1–3%, and CD 68 and Factor VIII about 5% of the total area of the valves (Fig. 1G).

**Figure 2:**
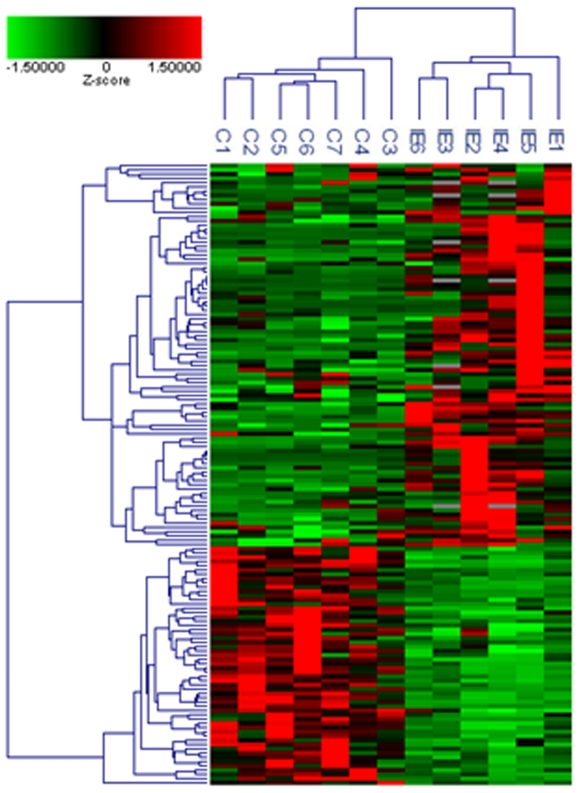
Hierarchical clustering analysis of transcriptional profiles of patients. Only 18,083 genes, which were expressed with a P-value <0.01 and a CV >0.3 in at least one condition, were included in the analysis. Data were converted to z-score prior gene and sample classifications by unsupervised hierarchic clustering using the average linkage method and Cosine correlation coefficient as the distance metric. The hierarchical clustering of a selection of 146 genes differentially expressed by IE and controls is showed, with a colour gradient (Z-score) from green (down-regulation) to red (up-regulation).

Using whole genome approach, we investigated the transcriptional profiles of cardiac valves from 5 patients with IE, caused by either *S. aureus* or *Streptococcus* sp. and 7 controls with uninfected valvular heart diseases. In addition, we included one cardiac valve from a patient with suspected IE, i.e. exhibiting histological features of IE (low expression of CD15 and CD68, and high expression of Factor VIII, Fig. 1G) but negative blood culture. Among the 33,492 sequences (41,000 reporters) of the microarray, only annotated genes (18,083 genes) and those with P-value_pres_ <0.01 and a coefficient variation >0.3 (11,404 genes) were used for global clustering analysis and differential analysis between IE and controls; genes with P-value_diff_ <0.01 and an absolute fold change (FC) >3.0 were considered as differentially expressed. The IE signature consisted of 146 genes, 89 being up-regulated with FC ranging from 3.1 to 58.9 (Table S1), and 57 genes down-modulated with FC ranging from −3.1 to −14.9 (Table S2). The transcriptional profiles of patients were organized in a common cluster placed on a branch distinct from controls (Fig. 2). Interestingly, the transcriptional profile of the cardiac valve from the patient with suspected IE (patient IE6) clustered with those of IE patients (Fig. 2). This suggests that the gene expression profile of heart valves enables to discriminate patients with IE from controls. It is noteworthy that the transcriptional programme of IE valves reflected the level of leucocyte infiltration. We identified the neutrophil signature (27 genes) but not the lymphocyte signature (Fig. S1) previously reported in isolated cells [8].

**Figure 3:**
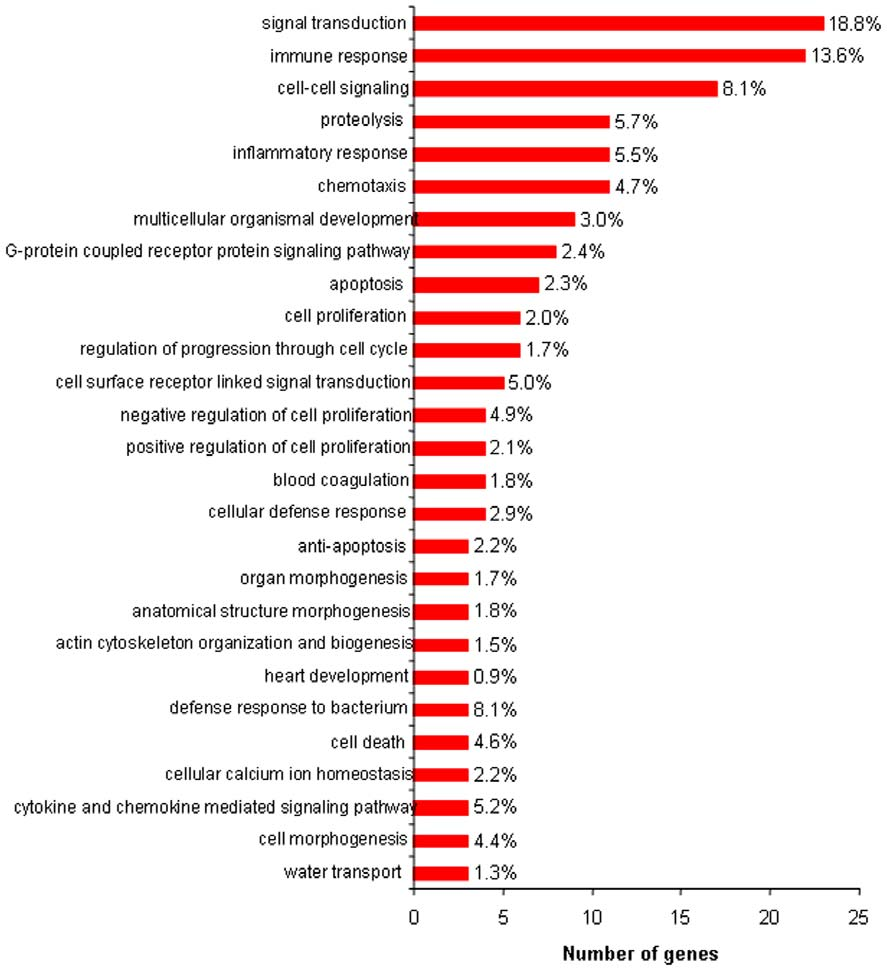
GO annotation of modulated genes. Differentially expressed genes in IE were subjected to GO annotation to identify the corresponding biological process. The major biological processes are shown, including the number of genes for each of the processes, and the percentage of differentially expressed annotated genes. GO redundancy is due to the involvement of individual genes in multiple biological processes.

We then analyzed IE-associated genes by using the Gene Ontology (GO) annotation and the resulting functional classification. We found enriched GO terms related to immune response, inflammatory response, chemotaxis, proteolysis, cellular defence response, defence response to bacteria, cell-cell signalling, calcium homeostasis and positive regulation of cell proliferation (Fig. 3). They may be classified in four functional groups: immune response, structural organization or remodelling, proliferation/death, and metabolism/miscellaneous (Table S1). This is consistent with mechanisms described in animal models of IE, i.e., recruitment of cells in vegetations, remodelling of endocardium tissue and neo-angiogenesis [4]. The down-modulated genes in IE valves mainly belong to structural organization or remodeling (n = 9), proliferation/death (n = 7), metabolism (n = 7) and miscellaneous (n = 31) functional groups. No gene involved in inflammatory, immune and defence responses to bacteria was down-modulated (Table S2).

An important step in vegetation genesis during experimental IE is leucocyte attraction and angiogenesis [4]. In this study, we showed a large number of up-regulated genes involved in chemotaxis with FC ranging from 3.9 to 58.9 (Table S1). These genes encode both CXC chemokines (CXCL1, CXCL4, CXCL5, CXCL6, CXCL7, CXCL13) involved in neutrophil recruitment, and CC chemokines (CCL7, CCL8, CCL13, CCL18, CCL20, CCL23) involved in the trafficking of myeloid and T cells [9]. The up-regulation of CCL13, CXCL1, CXCL5 and CXCL6 genes was confirmed by qRT-PCR in these patients and another set of 9 patients (Fig. S2). CXCL1, CXCL4 and CXCL5 have pleiotropic functions including neutrophil activation and adhesion of monocytes to endothelial cells [10]; CXCL6, largely produced by mesenchymal cells in response to inflammatory mediators, is cleaved by group A *Streptococcus* protease, which abrogates its activity [11]; CCL13 is involved in the migration of monocytes, T cells and eosinophils [12]; CCL20 mediates the recruitment of T cells and dendritic cells, and is expressed by Th17 cells [13]; CCL18 is associated with M2 polarization of macrophages [14]. Altogether, this large panel of up-regulated genes encoding chemokines suggests that the attraction of leucocytes to the vegetation, including dendritic cells as recently reported [15], is one crucial step of vegetation development. Chemokines, such as CXCL1, CXCL6 and CXCL16, are involved in angiogenesis [9], and the genes encoding these chemokines were up-regulated in patients. CXCL16 is also expressed in valves from patients with rheumatic or atherosclerotic diseases, and may be necessary for the recruitment of CD8^+^ T cells during inflammatory valvular heart disease [16].

Tissue remodeling is a prerequisite for vegetation development by promoting leucocyte transmigration. In vitro experiments show that recruited monocytes in valvular lesions contribute to the formation of vegetations by producing tissue factor and enhancing fibrin deposition [17]. Experimental models of IE using *Staphylococcus epidermidis*- or *S. aureus*-infected rabbits reveal the production of tissue factor by monocytes infiltrating vegetations [4], [18]. Moreover, there is a correlation between the numbers of infiltrating monocytes and bacteria inside the vegetation [18]. Among the 16 genes involved in tissue remodelling that were modulated in patients, 9 belonging to proteolysis process were up-regulated. They included a disintegrin and metalloproteinase (MMP12), also known as ADAM12 (Table S1). The expression of MMP12 was assessed by RT-PCR (Fig. S3) and immunohistochemistry (Fig. S4). MMP-12 transcripts were higher in IE valves than in controls, and the MMP-12 molecule was expressed in macrophages infiltrating IE valves, reflecting the inflammatory response of IE valves. MMPs, a family of endopeptidases that are secreted as latent zymogens, are involved in the pathogenesis of vascular disorders [19] and the remodelling of valvular tissues in endocarditis and degenerative valvular diseases [20]. The activities of MMPs are controlled at three distinct levels: gene expression, activation of the proenzyme forms of the MMPs, and inhibition of activity by complexing with their specific tissue inhibitors (TIMPs) [21]. Interestingly, TIMP-3 was down-modulated in IE patients (Table S2 and Fig. S3). Combined with increased expression of MMP-12, it may exacerbate valvular inflammation. As plasmin and thrombin activate MMPs, it is likely that the coagulation-fibrinolysis system can stimulate the MMPs leading to valvular destruction and embolization.

An anti-infectious response was also identified in IE valves (Table S1). First, it included the up-regulated expression of genes encoding chemokines, including CXCL1, CXCL13, CCL13, CCL18 and CCL20, with antimicrobial activity against bacteria such as *S. aureus* or *S. pyogenes* [22]. Second, it included members of C-type lectin domain superfamily, such as CLEC4D (with the highest FC), macrophage receptor with collagenous structure (MARCO, see Fig. S3) and leukocyte immunoglobulin-like receptors (LILR) B2 and B5. C-type lectins, characterized by the presence of one or more C-type domains, are able to bind microorganisms, activate complement and exert direct antimicrobial activity. These sensors of pathogens and cellular damage are mainly expressed by myeloid cells found in IE valves [23]. Third, the over-expression of the gene encoding granzyme B (see Fig. S3), a protease of cytotoxic CD8^+^ T cells, may be related to the presence of CD8^+^ T cells close to endothelial cells in inflammatory valve diseases [16] and in IE (our results). Fourth, three inflammatory cytokines, namely interleukin (IL)-1α (see Fig. S3), IL-24 and TNFSF14, were over-expressed in IE. Fifth, two members of the aquaglyceroporin membrane channel family classification were modulated in IE. The expression of aquaporin (AQP)-9 gene was increased by 18 fold in IE valves (see Fig. S3) and that of AQP-7 gene was down-modulated (Table S2). AQPs are cell membrane-embedded proteins that facilitate movement of water by increasing membrane water permeability and water flux in response to osmotic gradients [24]. They differ in their transcriptional regulation [25]. The AQP family can be divided into two groups on the basis of their permeability characteristics. Most members of the first group (AQP) are only permeated by water. Members of the second group (aquaglyceroporins), which includes AQP7 and AQP9, are permeated by water to varying degrees and other small solutes such as glycerol [24]. AQPs are involved in many pathological processes including myocardial oedema related to the ischemia-reperfusion phenomena [26]. AQP-7 is preferentially associated with adipose cardiac tissue [27], [28]. At our knowledge, the potential implication of AQPs in heart valve diseases has never been reported. However, recent works identified the ubiquitous expression of AQP water channels in the endothelial cells of most organs and their implications in the water movement across the capillary bed into the interstitial space [29]. As we found that AQP9 was expressed in endothelial cells lining the lumen of neo-vessels as demonstrated by immunohistochemistry (Fig. S4), we can speculate that the high increase in AQP9 expression observed during IE might be associated with the neo-vascularization of the infected valve leading to tissue oedema secondary to the inflammatory process.

**Figure 4:**
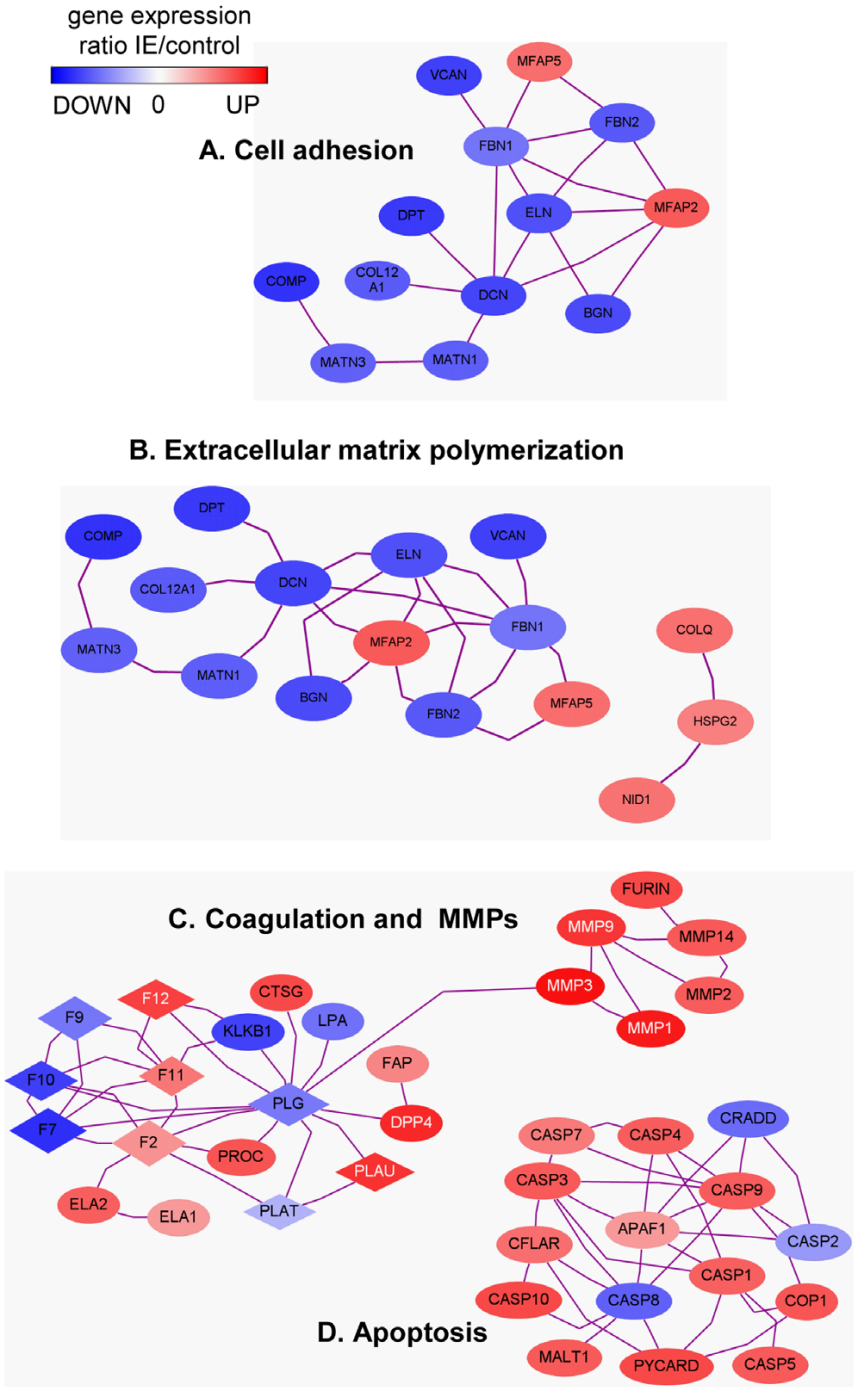
Network analysis in IE. Differentially expressed genes in IE were subjected to GO annotation according cell adhesion (A), extracellular matrix polymerization (B), proteolysis (C) and caspase (D) networks. Only entities with binding partners are represented. Note that the proteolysis network included coagulation and MMP pathways that were connected whereas caspase pathway was isolated.

Functional networks were identified using GO classification and web-based entry tool. We selected only interactions in which at least 2 references can be extracted from the literature. The first network associated with cell adhesion consisted of 13 genes mostly down-regulated (11 of 13 genes) (Fig. 4A). The second network was associated with extracellular matrix polymerization (Fig. 4B). The third network consisted of coagulation-related genes and related MMP genes (Fig. 4C). The coagulation network included 10 up-regulated genes such as PLAU, CTSG (cathepsin G) and F12 (coagulation factor 12), and 7 down-modulated genes such as KLKB1 (kallikrein B1), FVII and X (coagulation factor VII and X). Moreover, 5 genes encoding MMPs and that encoding furin, an endoprotease, were up-regulated in the network. This network analysis is consistent with the expression of coagulation factors and isolated MMPs in pathological valves [20], [30]. Moreover, our analysis revealed an apoptosis network, including 10 caspases and 5 related molecules (Fig. 4D). The caspase-8 gene was down-modulated and the caspase-9 gene was over-expressed in IE patients. As they are involved in the extrinsic and intrinsic apoptotic ways [31], respectively, it is likely that only caspase-9 is involved in IE. This is in accordance with recent data that showed activated caspases in leucocytes from patients with valvular diseases [32]. In an animal model, scintigraphic measurement of apoptosis confirms the presence of apoptotic cells in the vicinity of vegetations [33].

**Figure 5:**
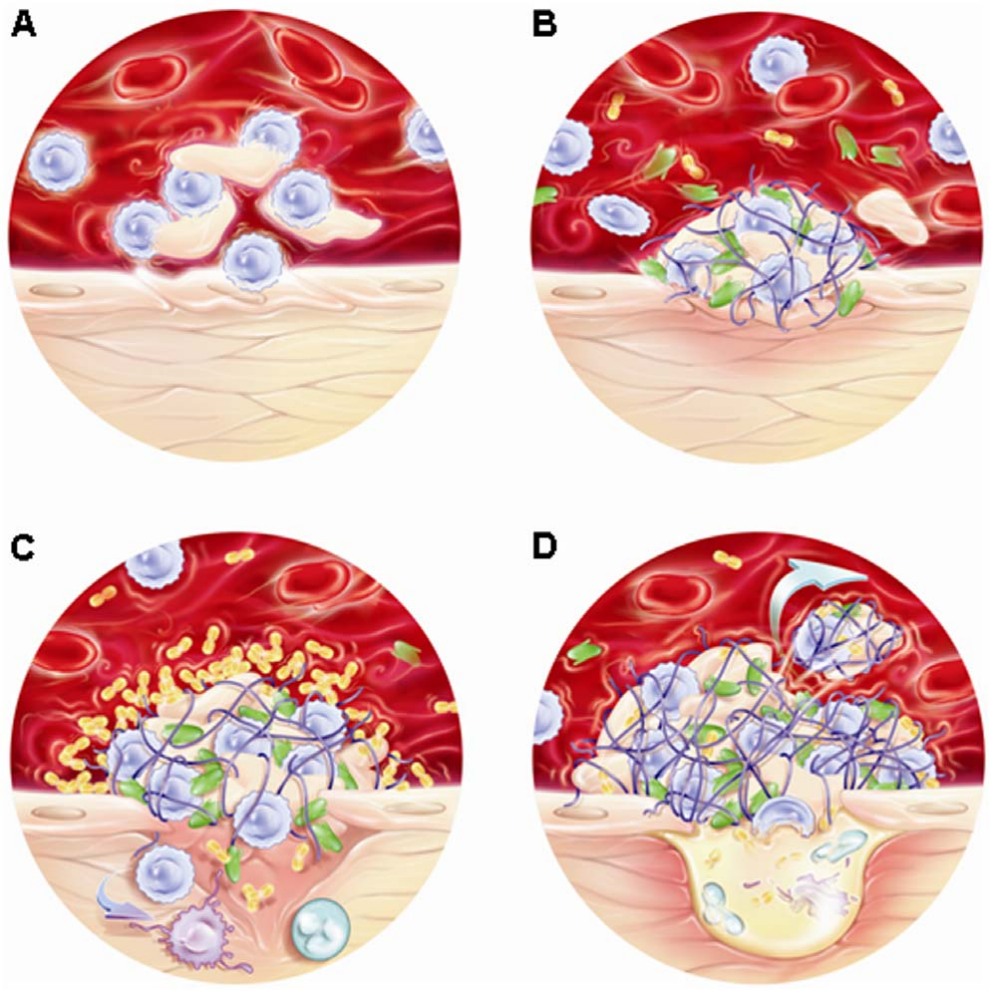
Putative scenario for IE. The natural history of IE may be decomposed in successive steps including cell apoptosis that may be promoted by blood turbulence in the vicinity of valve lesion (A), procoagulant activity that results in fibrin and platelet deposition (B), bacterial colonization and chemoattraction of neutrophils increasing vegetation size (C), tissue remodelling and neoangiogenesis leading to the functional destruction of the valve (D). At this stage, the situation is irreversible and cardiac surgery is necessary.

The whole genome analysis of cardiac valve transcriptome led us to propose a scenario for IE pathophysiology that relies gene expression and tissue lesions. In patients with valve defect, the blood flow is turbulent rather than linear [34] and this leads to the apoptosis of cells projected on cardiac valve (apoptosis network) (Fig. 5A). This is the initial event leading to tissue remodelling (extracellular matrix polymerization and coagulation networks) generating primary aseptic clot (Fig. 5B). Modified tissues enable microbial colonization and the recruitment of neutrophils and monocytes (chemotaxis) leading to the constitution of the vegetation (Fig. 5C). Then, tissue remodelling and neo-angiogenesis components (proteolysis and chemotaxis) destroy progressively the valve generating a risk of cardiac embolization and cardiac insufficiency (Fig. 5D). Based on this scenario, we suspected that the detection of apoptotic cells in the blood of patients with valve lesion may be associated with an increased risk of IE. This may be of great clinical impact to identify patients at risk. The prognosis value of circulating levels of MMP-12 and AQP-9 should be investigated in patients.

## Materials and Methods

### Patients

Ethics statement. Informed and written consent was obtained from each subject and the study was approved by the Ethics Committee of the Université de la Méditerranée.

The study consisted of a retrospective series of heart valve samples from 15 Caucasian patients hospitalized because of clinical suspicion of IE [35]. The15 tissue specimens analyzed in the present study were randomly selected from all the patients who underwent surgery for a first episode of left-sided native valve endocarditis between January 2006 and January 2008. The diagnosis of IE was based on the modified Duke's criteria [36] that include clinical data, blood cultures, immunohistochemical analyses of cardiac valves and molecular detection of organisms in blood and cardiac valves. Among IE patients, 5 patients (3 males and 2 females, median age: 59.6 years, range: 45–77 years) suffered from *S. aureus* infection, 9 patients (8 males, 1 female, median age: 52.5 years, range: 25–75 years) from IE due to *Streptococcus* sp. and 1 patient had an uncertain IE (one male, age: 59 years). As controls, we used cardiac valve samples from 12 patients (10 males and 2 females, median age: 61.8 years, range: 31–83 years) with pre-existing cardiac diseases. Thirteen samples (7 controls, 5 IE, 1 suspected EI) were investigated with microarrays and all the samples were analyzed by real-time quantitative RT-PCR.

### Histological Analysis

Valve tissue samples were fixed in formalin, embedded in paraffin, cut to 3 µm in thickness and stained with haematoxylin-eosin-saffron using routine methods. The immunohistological procedure, in which a peroxidase-based method was used, is described elsewhere [37]. Paraffin sections were stained with the ready-to-use CD15 (BD Biosciences, Le Pont de Claix, France), CD68 and Factor VIII (Dako, Trappes, France). The CD15-, CD68- and Factor VIII-positive surfaces were analyzed in tissue samples by quantitative image analysis, as described elsewhere [38]. In brief, immunohistological images were digitized and the image analyzer Samba 2005 (Samba Technologies, Alcatel TITN, Grenoble, France), which provides a visual control of analysis, allows the calculation of the percentage of the total surface area covered by the specific marker. For each set of observations, the surfaces of 10 randomly chosen areas were studied at a magnification of ×100, and the surface areas covered by neutrophils, macrophages and endothelial cells, respectively, were measured. The results are expressed in mean, with the minimum and the maximal values. MMP-12 and AQP9 were detected according a similar procedure with specific antibodies purchased from R&D Systems (Lille, France) and Chemicon (Millipore, Mosheim, France), respectively.

### Total RNA Extraction

Cardiac valve samples were collected in RNase-free tubes containing RNA*later*, a stabilization reagent. Tissue (10 mg) is then lysed with the TissueLyzer (Qiagen, Hilden, Germany) and total RNA was extracted using the RNeasy Mini kit (Qiagen) as previously described [32]. DNase treatment was performed with the DNase RNase-free set (Qiagen). RNA quality and quantity were assessed with the 2100 Bioanalyzer (Agilent Technologies, Santa Clara, California, USA) and the NanoDrop Spectrophotometer (NanoDrop Technologies, Wilmington, Delaware, USA).

### Microarray Experiments

Human 4×44k 60-mer oligonucleotide microarray slides (Agilent Technologies) and one-color experimental design were used as recently described [39]. All experiments were performed in an ozone-free area to ensure the stability of the cyanine 3 (Cy3). Sample labelling and hybridization were performed according to protocols specified by the manufacturer (One-Color Microarray-Based Gene Expression Analysis). Briefly, 300 ng of total RNA and Cy3-labeled CTP fluorescent dyes were used to generate fluorescent cRNA with Low RNA Input Fluorescent Amplification Kit (Agilent Technologies). The dye-incorporation ratio and the cRNA quantity were determined using the Nanodrop spectrophotometer. For hybridization, 1.65 µg of Cy3-labeled cRNA was added on microarray slide for 17 hours using the Hybridization Oven kit procedure provided by Agilent Technologies. Slides were then washed, dried, and scanned at 5 µm resolution with a G2505B DNA microarray scanner (Agilent Technologies).

### Analysis of Microarray Experiments

Image analysis and intra-array signal correction (one-color analysis default setting) were performed using Feature Extractor Software A.9.1.3 (Agilent Technologies). The use of Rosetta error model is suitable to improve the analysis of experiments with a small number of samples. Data processing, analysis and visualizing were performed using Resolver software 7.1 (Rosetta Inpharmatics, Seattle, WA) and its intensity error model pipeline optimized for the gene expression analysis of microarrays. The intensity error model and its applications have been detailed by Weng *et al.* [40]. Briefly, reporter mapping to genes was computed by performing a squeeze operation that created intensity profiles by combining replicates while applying error weighting. To know whether a gene was present in transcripts a hypothesis test was used. Negative control sequences were used to estimate the parameters of the null distribution and P-value (P-value_press_) was calculated for each particular sequence. When P-value_pres_ <0.01, we rejected the null hypothesis and accepted the alternative hypothesis that the sequence transcript was present in the microarray. Before error-weighted combining of samples in IE vs. controls, an inter-array global normalization was performed using the average of intensities from all positive or present genes. Discrimination between sample groups (IE vs. controls) was studied using an error-model-based hypothesis test. The null hypothesis was that the gene is not differentially expressed. A differential expression of P-value (P-value_pres_) involving parameters of the error model was computed for each particular sequence to compare gene expression between two category groups. In addition, FC (ratios in log 10 scale) were also computed using an error-weighted ratio combination method. For P-value_diff_ <0.01 and absolute FC >3.0, the gene was considered as differentially expressed.

The GO viewer tool was used to calculate P-value for each GO term. An exact hypergeometric distribution allowed the comparison of the frequencies of individual GO terms within the IE signature with the frequencies of those terms on the entire microarray (P<0.05 was considered to be significant). In order to increase meaningfulness and clarity, the output GO term list was filtered to only keep GO terms constituted of at least 5 genes belonging to the IE signature. Significant GO terms are separated in 3 categories: cell component, molecular function or biological process. For each GO term category, a 2D-cluster was performed considering significant GO terms vs. sample groups (controls and IE). We classified the GO terms by unsupervised hierarchic clustering, using the average linkage method and Cosine correlation coefficient as the distance metric whereas the sample group order was supervised. All data were entered in the ArrayExpress database following the MIAME procedure [41] and can be retrieved using the accession number E-MEXP-1334. Functional classification was determined using numerous databases: DAVID Bioinformatics Resources 2008 (http://david.abcc.ncifcrf.gov/), Online Medelian Inheritance in Man (http://www.ncbi.nlm.nih.gov/sites/entrez?db=OMIM&TabCmd=Limits), SOURCE (http://smd.stanford.edu/cgi-bin/source/sourceSearch), gand Babelomics Fatigo+ (http://babelomics2.bioinfo.cipf.es/fatigoplus/cgi-bin/fatigoplus.cgi).

The connection between genes was studied using networks generated by PathwayStudio™ (Ariadne Genomics) as recently described [39]. Briefly, networks were built by connecting entities with binding relations stored in the ResNet 6 mammal database. Ratio data from microarray experiments were used to colourized entities. Red colour corresponded to up-regulated genes in valves from IE patients compared to valves from controls and blue colour to down-regulated genes.

### Reverse Transcription and Quantitative Real-Time PCR (qRT-PCR)

The cDNA synthesis was carried out with 10 ng of total RNA, oligo(dT) primer and M-MLV reverse transcriptase (Invitrogen, Cergy Pontoise, France) according to the manufacturer's protocol. PCR was performed using the Light Cycler from Roche Diagnostics (Meylan, France). Briefly, amplification was conducted in a 20 µl volume using Syber Green PCR Maxter mixture (Roche Diagnostics), 2 µl of template cDNA, 1 µl (10 pmol) each of forward and reverse gene-specific primers, 2 µl of 3 mM MgCl_2_ and 12 µl H_2_O. The primers (Table S3) were designed using the primer3 tool (http://frodo.wi.mit.edu/cgi-bin/primer3/primer3_www.cgi). RT was omitted in negative controls. The FC in target gene cDNA relative to the β-actin endogenous control was determined as follows: FC = 2^−ΔΔCt^, where ΔΔCt = (CtTarget - CtActin)_IE_ - (CtTarget - CtActin)_controls_. Ct values were defined as the number of cycles for which the fluorescence signals were detected [42]. Results from the 14 patients with IE and the 12 controls are represented as FC median with 25 and 75 percentile distribution, and minimum and differences were considered statistically significant at a value of P<0.05.

## Supporting Information

Figure S1 Cellular signatures of cardiac valves. The list of genes corresponding to neutrophil (A) and lymphocyte (B) signatures is shown. Red colour corresponds to up-regulated genes, blue colour to down-regulated genes and grey colour to unexpressed genes in IE patients compared with controls.
(0.16 MB TIF)

Figure S2 Modulation of 4 genes encoding chemokines using qRT-PCR. The expression levels of 4 genes found up-regulated by microarray were determined by qRT-PCR and normalized with the β-actin gene. Results of cardiac valves from 12 controls (C) and 14 IE patients are represented as median with 25 and 75 percentile distribution, and minimum and maximum values. *P<0.05.
(0.06 MB TIF)

Figure S3 Modulation of different genes using qRT-PCR. The expression levels of 6 genes found up-regulated and one down-regulated by microarray were determined by qRT-PCR and normalized with the β-actin gene. Results of cardiac valves from 12 controls (C) and 14 IE patients are represented as median with 25 and 75 percentile distribution, and minimum and maximum values. *P<0.05.
(0.06 MB TIF)

Figure S4 Immunodetection of MMP-12 and AQP9 in IE valves. Valve tissue samples from 3 IE patients were freezed and cut to 3 µm in thickness. MMP-12 and AQP9 were revealed using specific antibodies (1/100 and 1/200 dilutions, respectively) and secondary antibodies coupled with peroxidase. Magnification: ×400.
(4.64 MB TIF)

Table S1 Genes up-regulated in IE
(0.18 MB DOC)

Table S2 Genes down-modulated in IE
(0.15 MB DOC)

Table S3 Primers used for qRT-PCR
(0.03 MB DOC)
