# Supplementary figures and images for: The Transcriptional Programme of Human Heart Valves Reveals the Natural History of Infective Endocarditis
Source: PLoS One. 2010 Jan 28;5(1):e8939. doi: 10.1371/journal.pone.0008939 (PMC2812508; doi:10.1371/journal.pone.0008939)

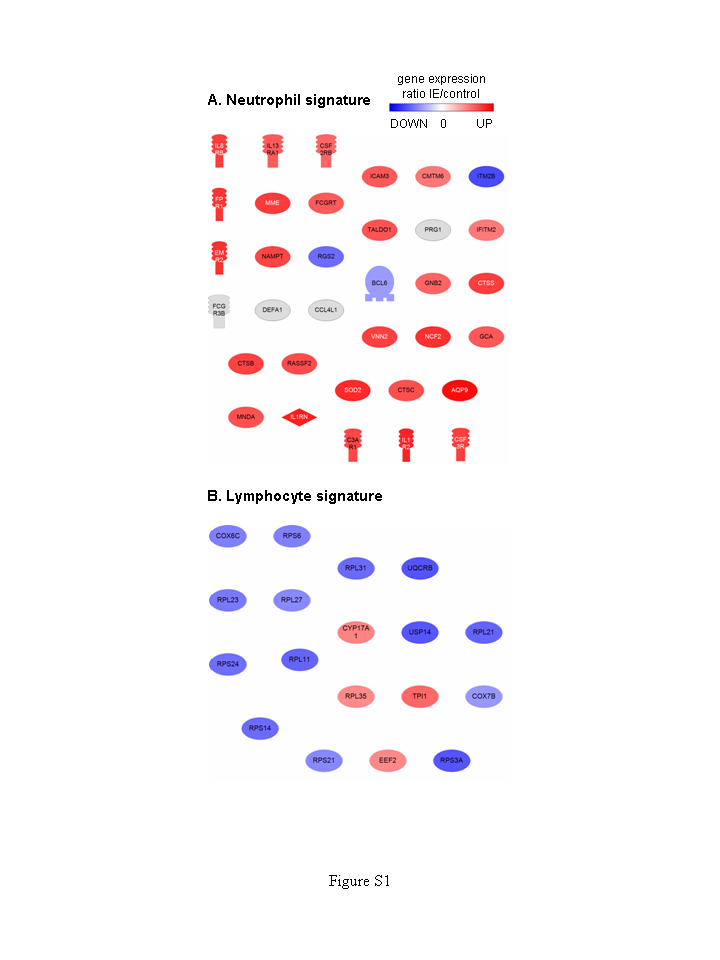

Supplement: Figure S1 — Cellular signatures of cardiac valves. The list of genes corresponding to neutrophil (A) and lymphocyte (B) signatures is shown. Red colour corresponds to up-regulated genes, blue colour to down-regulated genes and grey colour to unexpressed genes in IE patients compared with controls. (0.16 MB TIF) [file pone.0008939.s001.tif]

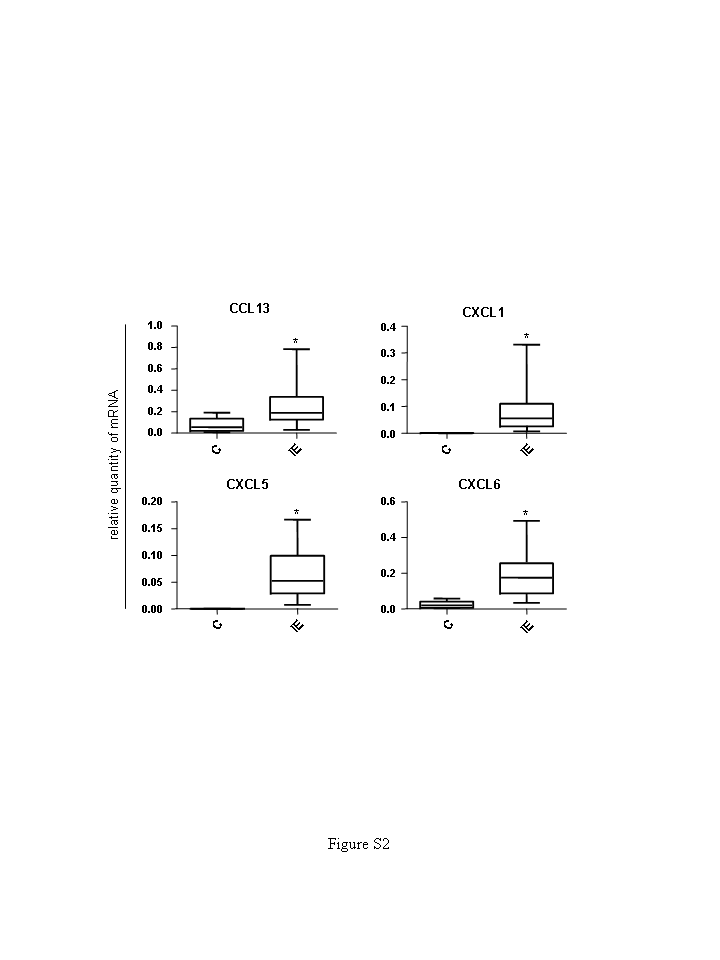

Supplement: Figure S2 — Modulation of 4 genes encoding chemokines using qRT-PCR. The expression levels of 4 genes found up-regulated by microarray were determined by qRT-PCR and normalized with the β-actin gene. Results of cardiac valves from 12 controls (C) and 14 IE patients are represented as median with 25 and 75 percentile distribution, and minimum and maximum values. *P<0.05. (0.06 MB TIF) [file pone.0008939.s002.tif]

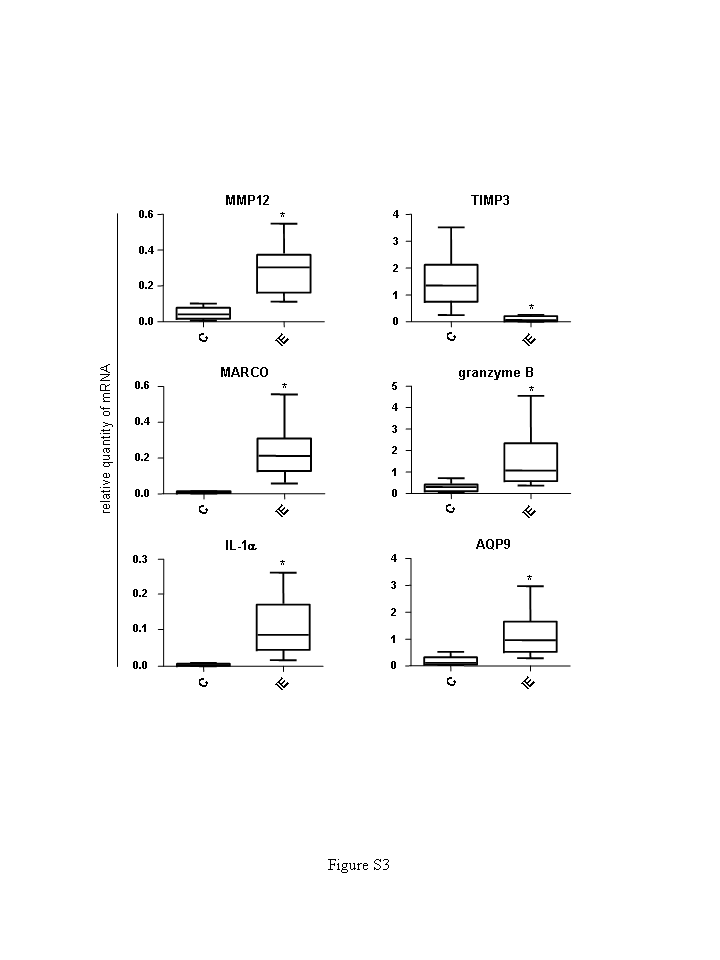

Supplement: Figure S3 — Modulation of different genes using qRT-PCR. The expression levels of 6 genes found up-regulated and one down-regulated by microarray were determined by qRT-PCR and normalized with the β-actin gene. Results of cardiac valves from 12 controls (C) and 14 IE patients are represented as median with 25 and 75 percentile distribution, and minimum and maximum values. *P<0.05. (0.06 MB TIF) [file pone.0008939.s003.tif]

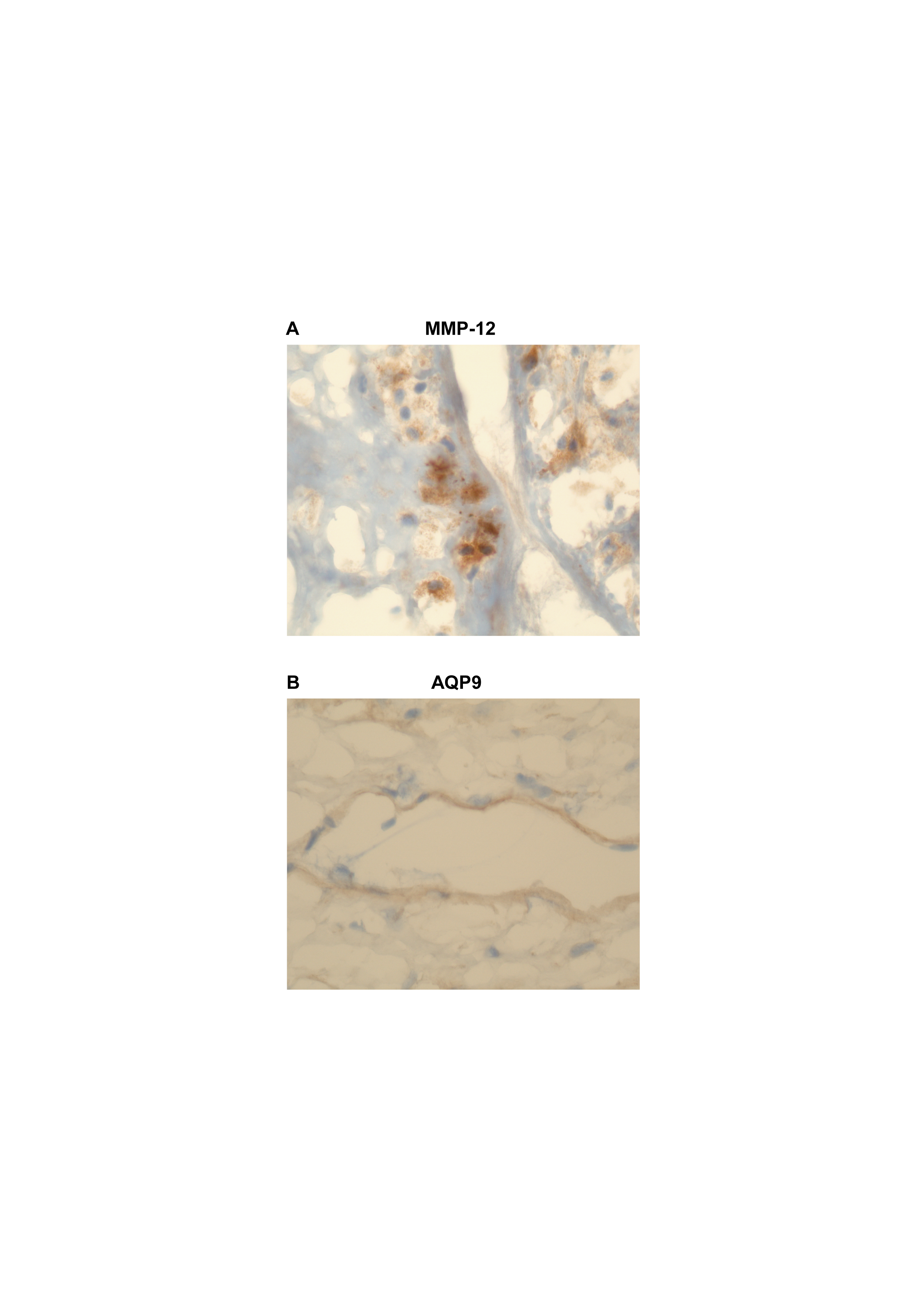

Supplement: Figure S4 — Immunodetection of MMP-12 and AQP9 in IE valves. Valve tissue samples from 3 IE patients were freezed and cut to 3 µm in thickness. MMP-12 and AQP9 were revealed using specific antibodies (1/100 and 1/200 dilutions, respectively) and secondary antibodies coupled with peroxidase. Magnification: ×400. (4.64 MB TIF) [file pone.0008939.s004.tif]
