# Supplementary material for: The Transcriptional Programme of Human Heart Valves Reveals the Natural History of Infective Endocarditis
Source: PLoS One. 2010 Jan 28;5(1):e8939. doi: 10.1371/journal.pone.0008939 (PMC2812508; doi:10.1371/journal.pone.0008939)
Supplement: Table S1 — Genes up-regulated in IE (0.18 MB DOC) [file pone.0008939.s005.doc]

**Table S1. Genes up-regulated in IE**

| **Sequence description** | **Gene symbol** | **Accession #** | **Gene ontology** | **FC** | **Classification** |
| --- | --- | --- | --- | --- | --- |
| chemokine (C-C motif) ligand 13 | CCL13 | NM_005408 | chemotaxis | 9.0 | immune response |
| chemokine (C-C motif) ligand 18 | CCL18 | NM_002988 | chemotaxis | 13.9 | immune response |
| chemokine (C-C motif) ligand 20 | CCL20 | NM_004591 | chemotaxis | 17.7 | immune response |
| chemokine (C-C motif) ligand 7 | CCL7 | NM_006273 | chemotaxis | 55.1 | immune response |
| chemokine (C-C motif) ligand 8. | CCL8, MCP2 | NM_005623 | chemotaxis | 3.9 | immune response |
| chemokine (C-C motif) ligand 23. | CCL23 | NM_005064 | chemotaxis | 7.6 | immune response |
| chemokine (C-X-C motif) ligand 1 | CXCL1 | NM_001511 | chemotaxis | 58.9 | immune response |
| chemokine (C-X-C motif) ligand 13 | CXCL13 | NM_006419 | chemotaxis | 14.2 | immune response |
| chemokine (C-X-C motif) ligand 5 | CXCL5 | NM_002994 | chemotaxis | 12.8 | immune response |
| chemokine (C-X-C motif) ligand 6 | CXCL6 | NM_002993 | chemotaxis | 31.6 | immune response |
| chemokine (C-X-C motif) ligand 4 | PF4/CXCL4 | NM_002619 | chemotaxis | 6.4 | immune response |
| chemokine (C-X-C motif) ligand 7 | PPBP/CXCL7 | NM_002704 | chemotaxis | 29.0 | immune response |
| plasminogen activator, urokinase | PLAU (u-PA) | NM_002658 | chemotaxis / proteolysis | 4.1 | immune response |
| C-type lectin domain family 4, member D | CLEC4D | NM_080387 | immune response | 35.1 | immune response |
| C-type lectin superfamily 4, member G | CLEC4G | NM_198492 | sugar binding | 8.1 | immune response |
| C-type lectin domain family 5, member A | CLEC5A | NM_013252 | cellular defense response | 7.7 | immune response |
| C-type lectin domain family 7, member A | CLEC7A | NM_197947 | inflammatory response | 6.7 | immune response |
| tumor necrosis factor, member 14 | TNFSF14 | NM_003807 | immune response | 7.3 | immune response |
| epiregulin | EREG | NM_001432 | cytokine and chemiokine mediated signaling pathway / organ morphogenesis | 9.5 | immune response |
| bradykinin receptor B1 | BDKRB1 | NM_000710 | inflammatory response | 11.3 | immune response |
| heparanase | HPSE | NM_006665 | inflammatory response | 6.1 | immune response |
| macrophage receptor collagenous structure | MARCO | NM_006770 | cell surface receptor- signal transduction | 17.2 | immune response |
| gremlin 1, cysteine knot superfamily | GREM1 | NM_013372 | cytokine activity | 16.8 | immune response |
| colony stimulating factor 3 | CSF3,GCSF | NM_000759 | cytokine and chemiokine mediated signaling pathway | 6.1 | immune response |
| complement factor properdin | CFP | NM_002621 | defense response to bacteria | 5.0 | immune response |
| leukocyte immunoglobulin-like receptor, subfamily B, member 2 | LILRB2 | NM_005874 | cellular defense response | 3.8 | immune response |
| granulysin | GNLY,519 | NM_006433 | defense response to bacteria | 4.0 | immune response |
| interleukin 2 receptor, alpha | IL2RA, CD25 | NM_000417 | immune response / cell proliferation | 4.6 | immune response |
| interleukin 1, alpha | IL1A | NM_000575 | inflammatory response | 16.5 | immune response |
| aquaporin 9 | AQP9 | NM_020980 | immune response / water transport | 17.9 | immune response |
| leukocyte immunoglobulin-like receptor, subfamily B, member 5 | LILRB5 | NM_006840 | immune response | 3.3 | immune response |
| T-cell, immune regulator 1, ATPase, H^+^ transporting, lysosomal V0 subunit A3 | TCIRG1 | NM_006019 | cellular defense response | 3.1 | immune response |
| interleukin 24 | IL24 | NM_006850 | immune response | 10.0 | immune response |
| CD70 molecule | CD70 | NM_001252 | immune response | 4.1 | immune response |
| *Homo sapiens* absent in melanoma 2 | AIM2 | NM_004833 | immune response | 9.1 | immune response |
| ADAM metallopeptidase domain 12 | ADAM12 | NM_021641 | proteolysis | 10.5 | structural organization or remodeling |
| granzyme B | GZMB | NM_004131 | proteolysis | 3.8 | structural organization or remodeling |
| kallikrein 1 | KLK1 | NM_002257 | proteolysis | 4.6 | structural organization or remodeling |
| carboxypeptidase X, member 1 | CPXM1 | NM_019609 | proteolysis | 3.7 | structural organization or remodeling |
| dipeptidase 2 | DPEP2 | NM_022355 | proteolysis | 3.9 | structural organization or remodeling |
| cathepsin H | CTSH | NM_148979 | proteolysis | 3.1 | structural organization or remodeling |
| matrix metallopeptidase 12. | MMP12 | NM_002426 | proteolysis | 9.0 | structural organization or remodeling |
| kallikrein-related peptidase 7. | KLK7 | NM_005046 | proteolysis | 6.7 | structural organization or remodeling |
| ADAM metallopeptidase with thrombospondin type 1 motif, 5. | ADAMTS5 | NM_007038 | proteolysis | 3.4 | structural organization or remodeling |
| transglutaminase 2 (C polypeptide, protein-glutamine-gamma-glutamyltransferase) | TGM2 | NM_198951 | positive regulation of inflammatory response / blood vessel remodeling | 3.5 | structural organization or remodeling |
| phosphatase and actin regulator 1 | PHACTR1 | NM_030948 | acting binding | 6.0 | structural organization or remodeling |
| sialic acid binding Ig-like lectin 12 | SIGLEC12 | NM_053003 | cell adhesion | 11.7 | structural organization or remodeling |
| Leukocyte specific transcript 1 | LST1 | NM_007161 | cell morphogenesis | 3.9 | structural organization or remodeling |
| spectrin, beta, non-erythrocytic 5 | SPTBN5 | NM_016642 | actin cytoskeleton organization and biogenesis | 3.5 | structural organization or remodeling |
| elastin microfibril interfacer 2 | EMILIN2 | NM_032048 | cell adhesion | 3.8 | structural organization or remodeling |
| podoplanin | PDPN | NM_006474 | cell morphogenesis | 4.6 | structural organization or remodeling |
| basonuclin 1 | BNC1 | NM_001717 | positive regulation of cell proliferation | 5.5 | proliferation / death |
| homeobox B4 | HOXB4 | NM_024015 | multicellular organismal development | 3.9 | proliferation / death |
| ets variant gene 7 (TEL2 oncogene) | ETV7 | NM_016135 | organ morphogenesis | 3.2 | proliferation / death |
| mitochondrial protein 18 kDa | MTP18 | NM_016498 | apoptosis | 4.3 | proliferation / death |
| activating transcription factor 5 | ATF5 | NM_012068 | regulation of progression through cell cycle | 3.1 | proliferation / death |
| tumor necrosis factor receptor superfamily, member 6b, decoy | TNFRSF6B | NM_032945 | apoptosis | 5.7 | proliferation / death |
| cation channel, sperm associated 1 | CATSPER1 | NM_053054 | multicellular organismal development | 7.8 | proliferation / death |
| tryptophan 2,3-dioxygenase | TDO2 | NM_005651 | metal ion binding | 28.8 | metabolism |
| B cell RAG associated protein | GALNAC4S-6ST | NM_015892 | hexose biosynthetic process | 3.0 | metabolism |
| aspartate beta-hydroxylase | ASPHD1 | NM_181718 | integral to membrane | 11.8 | metabolism |
| glycosyltransferase 1 domain containing 1 | GLT1D1 | NM_144669 | biosynthetic process | 6.3 | metabolism |
| solute carrier family 16, member 10 | SLC16A10 | NM_018593 | transporter activity | 10.8 | miscellaneous |
| solute carrier family 16, member 3 | SLC16A3 | NM_004207 | transporter activity | 6.3 | miscellaneous |
| solute carrier family 22 member 16 | SLC22A16 | NM_033125 | transporter activity | 4.3 | miscellaneous |
| solute carrier family 39 member 8 | SLC39A8 | NM_022154 | transporter activity | 5.1 | miscellaneous |
| solute carrier family 7, member 11 | SLC7A11 | NM_014331 | transporter activity | 8.1 | miscellaneous |
| syntaxin binding protein 2 | STXBP2 | NM_006949 | vesicle-mediated transport | 5.3 | miscellaneous |
| syntaxin 1A | STX1A | NM_004603 | vesicle-mediated transport | 3.4 | miscellaneous |
| asialoglycoprotein receptor 1 | ASGR1 | NM_001671 | receptor-mediated endocytosis | 4.4 | miscellaneous |
| seizure related 6-like 2, transcript variant 2 | SEZ6L2 | NM_201575 | receptor activity | 5.7 | miscellaneous |
| apolipoprotein B mRNA editing enzyme, catalytic polypeptide-like 3A | APOBEC3A | NM_145699 | metal ion binding | 12.0 | miscellaneous |
| carbonic anhydrase XII, transcript variant 1 | CA12 | NM_001218 | metal ion binding | 17.2 | miscellaneous |
|  |  |  |  |  | miscellaneous |
| PDZ and LIM domain 4 | PDLIM4 | NM_003687 | metal ion binding | 4.8 | miscellaneous |
| UDP-N-acetyl-alpha-D-galactosamine N-acetylgalactosaminyltransferase 14 (GalNAc-T14) | GALNT14 | NM_024572 | sugar binding | 6.1 | miscellaneous |
| glutamine-fructose-6-P transaminase 2 | GFPT2 | NM_005110 | sugar binding | 3.2 | miscellaneous |
| delta/notch-like EGF repeat containing | DNER | NM_139072 | signal transduction | 7.2 | miscellaneous |
| Ras association (RalGDS/AF-6) family 7 | RASSF7 | NM_003475 | signal transduction | 4.0 | miscellaneous |
| egf-like module containing, mucin-like, hormone receptor-like 3 | EMR3 | NM_032571 | signal transduction | 16.0 | miscellaneous |
| succinate receptor 1 | SUCNR1 | NM_033050 | signal transduction | 4.2 | miscellaneous |
| pyrimidinergic receptor P2Y, G-protein coupled, 6 | P2RY6 | NM_176798 | signal transduction | 3.6 | miscellaneous |
| growth differentiation factor 15 | GDF15 | NM_004864 | signal transduction | 3.4 | miscellaneous |
| cytidine deaminase | CDA | NM_001785 | cell surface receptor linked signal transduction | 3.1 | miscellaneous |
| myotubularin related protein 10 | MTMR10 | NM_017762 | phospholipid dephosphorylation | 4.0 | miscellaneous |
| zona pellucida glycoprotein 1 | ZP1 | NM_207341 | integral to membrane | 10.9 | miscellaneous |
| Nedd4 binding protein 3 | N4BP3 | NM_015111 | membrane | 4.7 | miscellaneous |
| organic solute transporter alpha | OSTalpha | NM_152672 | membrane | 7.6 | miscellaneous |
| transmembrane protein 132A, transcript | TMEM132A | NM_017870 | endoplasmic reticulum | 7.3 | miscellaneous |
| EPS8-like 1, transcript variant 1 | EPS8L1 | NM_133180 | unclassified | 5.3 | miscellaneous |
| EF-hand domain family, member D2 | EFHD2 | NM_024329 | unclassified | 3.6 | miscellaneous |
| FCH domain only 1 | FCHO1 | NM_015122 | unclassified | 3.6 | miscellaneous |
| RNA binding protein | FLJ20273 | NM_019027 | unclassified | 3.2 | miscellaneous |
| hexokinase 3, nuclear gene encoding mitochondrial protein | HK3 | NM_002115 | unclassified | 5.6 | miscellaneous |
| family with sequence similarity 20, member A | FAM20A | NM_017565 | unclassified | 3.4 | miscellaneous |
| collagen, type XXII, alpha 1. | COL22A1 | NM_152888 | unclassified | 5.6 | miscellaneous |
| cystatin A (stefin A) (CSTA), | CSTA | NM_005213 | unclassified | 4.0 | miscellaneous |
| oligonucleotide/oligosaccharide-binding fold containing 2A. | OBFC2A | NM_001031716 | unclassified | 3.1 | miscellaneous |
| meteorin, glial cell differentiation regulator-like | METRNL | NM_001004431 | unclassified | 3.3 | miscellaneous |
| histocompatibility (minor) 13 | HM13 | NM_178580 | unclassified | 3.4 | miscellaneous |
| pleckstrin and Sec7 domain containing 4 . | PSD4 | NM_012455 | unclassified | 4.4 | miscellaneous |
