# Supplementary material for: The Transcriptional Programme of Human Heart Valves Reveals the Natural History of Infective Endocarditis
Source: PLoS One. 2010 Jan 28;5(1):e8939. doi: 10.1371/journal.pone.0008939 (PMC2812508; doi:10.1371/journal.pone.0008939)
Supplement: Table S2 — Genes down-modulated in IE (0.15 MB DOC) [file pone.0008939.s006.doc]

**Table S2. Genes down-modulated in IE**

| **Sequence description** | **Gene symbol** | **Accession #** | **Gene ontology** | **FC** | **Classification** |
| --- | --- | --- | --- | --- | --- |
| carbonic anhydrase III, | CA3 | NM_005181 | cellular metabolic process | -11.06 | metabolism |
| Cytochrome b reductase 1 | CYBRD1 | NM_024843 | catalytic activity | -3.23 | metabolism |
| glutathione peroxidase 3 | GPX3 | NM_002084 | cellular metabolic process | -3.34 | metabolism |
| guanylate cyclase activator 2B | GUCA2B | NM_007102 | enzyme activator activity | -3.57 | metabolism |
| phospholipase C, eta 1 | PLCH1 | NM_014996 | metabolic process | -5.06 | metabolism |
| phosphoribosyl transferase | PRTFDC1 | NM_020200 | metabolic process | -4.36 | metabolism |
| protein tyrosine phosphatase, non-receptor type 13 | PTPN13 | NM_080685 | catalytic activity | -3.53 | metabolism |
| tropomodulin 1 | TMOD1 | NM_003275 | cytoskeleton | -5.13 | structural organization or remodeling |
| TIMP metallopeptidase inhibitor 3 | TIMP3 | NM_000362 | signal transduction | -5.78 | structural organization or remodeling |
| cadherin 19, type 2 | CDH19 | NM_021153 | cell adhesion | -7.92 | structural organization or remodeling |
| dishevelled associated activator of morphogenesis 1 | DAAM1 | NM_014992 | actin cytoskeleton organization and biogenesis | -3.67 | structural organization or remodeling |
| laminin, alpha 2 | LAMA2 | NM_000426 | regulation of cell migration | -3.1 | structural organization or remodeling |
| endothelin converting enzyme 2 | ECE2 | NM_014693 | proteolysis | -3.9 | structural organization or remodeling |
| corin, serine peptidase | CORIN | NM_006587 | proteolysis | -5.6 | structural organization or remodeling |
| calsyntenin 2 | CLSTN2 | NM_022131 | cell adhesion | -3.7 | structural organization or remodeling |
| transforming growth factor 2 | TGFB2 | NM_003238 | cell morphogenesis | -3.1 | structural organization or remodeling |
| deleted in bladder cancer 1 | DBC1 | NM_014618 | cell death | -4.9 | proliferation / death |
| fibroblast growth factor 14 | FGF14 | NM_175929 | cell-cell signaling | -5.46 | proliferation / death |
| sprouty homolog 2 (*Drosophila*) | SPRY2 | NM_005842 | organ morphogenesis | -4.91 | proliferation / death |
| GATA binding protein 4 | GATA4 | NM_002052 | heart development | -5.64 | proliferation / death |
| glycoprotein M6B | GPM6B | NM_001001996 | multicellular organismal development | -3.60 | proliferation / death |
| SRY (sex determining region Y) box-9 | SOX9 | NM_000346 | heart development | -3.8 | proliferation / death |
| meiosis-specific nuclear structural 1 | MNS1 | NM_018365 | meiosis | -3.3 | proliferation / death |
| immunoglobulin superfamily, member 3 | IGSF3 | NM_001542 | membrane | -3.56 | immune response |
| complement factor H-related 3 | CFHR3 | NM_021023 | complement activation | -4.27 | immune response |
| complement factor H | CFH | NM_001014975 | complement activation, alternative pathway | -3.9 | immune response |
| aquaporin 7 | AQP7 | NM_001170 | water transport | -4.0 | miscellaneous |
| solute carrier family 27-6 | SLC27A6 | NM_001017372 | transporter activity | -13.51 | miscellaneous |
| solute carrier family 6-1 | SLC6A1 | NM_003042 | transporter activity | -4.84 | miscellaneous |
| ATP-binding cassette, sub-family A | ABCA6 | NM_080284 | transport | -3.6 | miscellaneous |
| myosin VIIA and Rab interacting protein | MYRIP | NM_015460 | intracellular protein transport | -4.0 | miscellaneous |
| interferon regulatory factor 6 | IRF6 | NM_006147 | transcription factor activity | -4.41 | miscellaneous |
| sal-like 3 (*Drosophila*) | SALL3 | NM_171999 | transcription | -9.23 | miscellaneous |
| retinoic acid receptor, beta | RARB | NM_000965 | transcription | -3.6 | miscellaneous |
| zinc finger protein 566 | ZNF566 | NM_032838 | transcription | -3.85 | miscellaneous |
| zinc finger protein 519 | ZNF519 | NM_145287 | transcription | -3.2 | miscellaneous |
| G protein-coupled receptor 83 | GPR83 | NM_016540 | signal transduction | -14.86 | miscellaneous |
| coagulation factor II receptor | F2R | NM_001992 | signal transduction | -3.71 | miscellaneous |
| coagulation factor II receptor-like 1 | F2RL1 | NM_005242 | signal transduction | -5.74 | miscellaneous |
| coagulation factor II receptor-like 2 | F2RL2 | NM_004101 | signal transduction | -5.81 | miscellaneous |
| ankyrin 3, node of Ranvier | ANK3 | NM_001149 | signal transduction | -5.5 | miscellaneous |
| EPH receptor A3 | EPHA3 | NM_005233 | signal transduction | -7.4 | miscellaneous |
| PDZ domain containing RING | PDZRN4 | NM_013377 | metal ion binding | -6.7 | miscellaneous |
| patched domain containing 1 | PTCHD1 | NM_173495 | membrane | -8.78 | miscellaneous |
| ring finger protein 180 | RNF180 | NM_178532 | metal ion binding | -3.81 | miscellaneous |
| schlafen family member 13 | SLFN13 | NM_144682 | nucleotide binding | -4.97 | miscellaneous |
| phosphorylase kinase, gamma 1 | PHKG1 | NM_006213 | unclassified | -3.8 | miscellaneous |
| growth hormone regulated TBC protein 1 | GRTP1 | NM_024719 | regulation of Rab GTPase activity | -3.3 | miscellaneous |
| SET binding protein 1 | SETBP1 | NM_015559 | DNA binding | -3.2 | miscellaneous |
| sterile alpha motif domain containing 13 | SAMD13 | NM_001010971 | unclassified | -4.2 | miscellaneous |
| indolethylamine N-methyltransferase | INMT | NM_006774 | unclassified | -3.3 | miscellaneous |
| KPL2 protein | SPEF2 | NM_024867 | unclassified | -3.9 | miscellaneous |
| glycine-N-acyltransferase-like 2 | GLYATL2 | NM_145016 | unclassified | -3.3 | miscellaneous |
| ST8 alpha-N-acetyl-neuraminide alpha-2,8-sialyltransferase 1 | ST8SIA1 | NM_003034 | unclassified | -4.2 | miscellaneous |
| SLIT and NTRK-like family, member 4 | SLITRK4 | NM_173078 | unclassified | -3.2 | miscellaneous |
| family with sequence similarity 5 | FAM5C | NM_199051 | unclassified | -4.8 | miscellaneous |
| matrilin 2 | MATN2 | NM_030583 | unclassified | -5.7 | miscellaneous |
