# Supplementary material for: The Transcriptional Programme of Human Heart Valves Reveals the Natural History of Infective Endocarditis
Source: PLoS One. 2010 Jan 28;5(1):e8939. doi: 10.1371/journal.pone.0008939 (PMC2812508; doi:10.1371/journal.pone.0008939)
Supplement: Table S3 — Primers used for qRT-PCR (0.03 MB DOC) [file pone.0008939.s007.doc]

**Table S3.** Primers used for qRT-PCR

| Gene | Forward primer | Reverse primer |
| --- | --- | --- |
| CCL13 | 5'-aaaagctttcaacccccaggg-3' | 5'-aaatgtgagctttccggccc-3' |
| CXCL1 | 5'-agggaattcaccccaagaac-3' | 5'-taactatgggggatgcagga-3' |
| CXCL5 | 5'-tgtttacagaccacgcaagg-3' | 5'-ttgtttccaccgtccaaaat-3' |
| CXCL6 | 5'-gtcctgtctctgctgtgctg-3' | 5'-aacttgcttcccgttcttca-3' |
| MMP12 | 5'-aggcacaaagtgtgcagatg-3' | 5'-gtctgtgcttcctccaaagc-3' |
| TIMP3 | 5'-ctgacaggtcgcgtctatga-3' | 5'-agtcacaaagcaaggcaggt-3' |
| MARCO | 5'-caggtttggctggttttcct-3' | 5'-tctccttttcttccttgctgtc-3' |
| granzyme B | 5'-tccctgtgaaaagacccatc-3' | 5'-ttcgcactttcgatcttcct-3' |
| IL-1α  | 5'-gtaagctatggcccactcca-3' | 5'-gcctccaggtcatcatcagt-3' |
| AQP9 | 5'-gcactaataactcggcatcttg-3' | 5'-attctcttgctccctacaccact-3' |
| β-actin | 5’-GGAAATCGTGCGTGACATTA-3’ | 5’-AGGAAGGAAGGCTGGAAGAG-3’ |
